# Clinical Study of Nanofibrillar Cellulose Hydrogel Dressing for Skin Graft Donor Site Treatment

**DOI:** 10.1089/wound.2019.0982

**Published:** 2020-02-07

**Authors:** Raili Koivuniemi, Tiina Hakkarainen, Jasmi Kiiskinen, Mika Kosonen, Jyrki Vuola, Jussi Valtonen, Kari Luukko, Heli Kavola, Marjo Yliperttula

**Affiliations:** ^1^ Drug Research Program, Division of Pharmaceutical Biosciences, Faculty of Pharmacy, University of Helsinki, Helsinki, Finland.; ^2^ Department of Plastic Surgery, Helsinki Burn Centre, Helsinki University Hospital and University of Helsinki, Helsinki, Finland.; ^3^ UPM-Kymmene Corporation, Helsinki, Finland.

**Keywords:** nanofibrillar cellulose, wound dressing, skin graft donor site treatment, patient, clinical study

## Abstract

**Objective:** Skin graft donor site management is a concern particularly for elderly patients and patients with poor wound healing competence, and also because donor sites are a source of pain and discomfort. Although different types of dressings exist, there is no consensus regarding optimal dressing type on donor site care to promote healing, reduce pain, and improve patients' comfort.

**Approach:** This prospective, single-center clinical trial evaluated the performance of nanofibrillar cellulose (NFC) wound dressing (FibDex^®^ by UPM-Kymmene Corporation) for treatment of donor sites compared with a polylactide-based copolymer dressing. The study enrolled 24 patients requiring skin grafting with mean age of 49 ± 18. The primary outcome measure was wound healing time. Secondary outcomes, the epithelialization, subjective pain, the scar appearance assessed using the Patient and Observer Scar Assessment Scale (POSAS), and skin elasticity and transepidermal water loss (TEWL), were evaluated at 1 and 6 months postoperatively.

**Results:** No statistically significant differences were observed between NFC and copolymer dressings regarding wound healing time, epithelialization, experience of pain, or TEWL. Significant differences were observed in the POSAS results for thickness and vascularity in the Observer score, in the favor of NFC over copolymer dressing. Moreover, skin elasticity was significantly improved with NFC dressing in terms of viscoelasticity and elastic modulus at 1 month postoperatively.

**Innovation:** NFC dressing is a new, green sustainable product for wound treatment without animal or human-origin components.

**Conclusion:** NFC dressing provides efficient wound healing at skin graft donor sites and is comparable or even preferable compared with the copolymer dressing.

**Figure:**
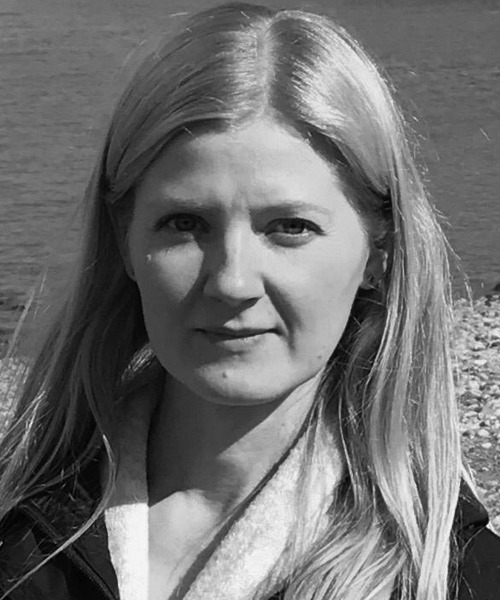
**Raili Koivuniemi, PhD**

## Introduction

The management of the donor site after split-thickness harvesting may be problematic due to delayed healing, especially in elderly patients, due to pain and discomfort at the donor site, or in patients with systemic comorbidities.^1,2^ A wide variety of dressings are available but no widely approved material exists for these wounds. Various dressings raise issues regarding low absorption to exudate, desiccation, frequent dressing changes, developing resistance to microbes, or price.^3^ An ideal wound dressing would promote re-epithelialization, provide a moist environment, prevent growth of micro-organisms, absorb exudate, and be transparent and comfortable for the patient and cost-effective and easy to apply.

Nanofibrillar cellulose (NFC) derived from wood with nanoscale diameter and microscale length is potentially a cost-efficient material to be used in pharmaceutical or biomedical applications. Having high aspect ratio, that is, a ratio of particle length to width and high elastic modulus, NFC can be easily modified into various forms, such as hydrogels, films, and aerogels.^4–6^ These physical and mechanical properties have made NFC useful as a drug and cell carrier, or cell scaffold.^7–10^ Further, the potential of NFC for wound treatment applications has recently been recognized based on its biocompatibility and ability to absorb and retain moisture.^11–14^

In comparison, bacterial cellulose (BC) and carboxymethyl cellulose (CMC) share many advantageous wound healing properties with NFC, such as biocompatibility and ability to absorb high contents of water so as to retain a moist environment.^15^ Specifically, these basic properties of BC, in addition to good permeability, resistance to degradation, and low solubility, are advantageous for tissue regeneration, and they have been demonstrated in clinical studies.^16^ In treatment of venous leg ulcers, BC-based wound dressing was found to create a moist, protective, and hypoxic environment for wound healing.^17^ Further, application of BC wound dressing to lower extremity ulcers have shown a remarkably shorter time of wound closure in comparison to standard care.^18^ In another study, BC wound dressing was evaluated as superior compared with a standard wound care regarding pain control, ease of use, and patient and nursing staff satisfaction.^19^ BC is synthesized in pure form^20^ by several bacterial species and has a similar chemical structure compared with NFC. However, their macromolecular properties differ, and BC has, for example, not only higher crystallization^21,22^ but also high fiber density that has been shown to limit cell infiltration.^23^ In addition, introduction of functional groups to enhance cell adhesion and biodegradability of the BC remains a challenge since microbial fermentation conditions are limiting and, therefore, restrict the introduction of many additive materials required to control porosity and BC nanofiber structure.^20,24^ CMC, on the other hand, is a cellulose derivative containing a large number of carboxymethyl groups on a cellulose backbone.^25^ However, it requires crosslinking to form a hydrogel structure, which often produces poisonous byproducts.^26,27^ CMC-based hydrofiber dressing has been introduced as costly, more painful, and not easy to use compared with other split-thickness skin graft donor site dressings.^28,29^

We have previously shown that NFC-based wound dressing is a promising material in clinical use for skin graft donor site treatment as it provides good attachment and adherence to the wound bed, and smooth automatic detachment after skin re-epithelialization.^30^ In this study, we aimed at evaluating more closely the effectiveness of NFC wound dressing in skin graft donor site treatment compared with a polylactide-based copolymer dressing, which we use as a standard treatment. The copolymer dressing is a synthetic and absorptive wound dressing consisting of dl-lactide, ɛ-caprolactone, and trimethylene carbonate that offers instant adaptability to the wound bed.^31^ Polylactids are a class of biodegradable polyesters that have been widely used in different biomedical applications due to their biocompatibility.^32^ The structure of the copolymer dressing is highly porous, which enables the moisture permeability and, thus, supports wound healing and re-epithelization, hindering the accumulation of wound exudate. Other advantages of using the copolymer dressing in wound treatment are its transparency and ability to detach from the wound site as the wound heals.^33^

We hypothesized that the mean healing time of wounds treated with NFC dressing would be comparable to wounds treated with the copolymer dressing. Further, we speculated that NFC dressing would serve as an effective wound dressing in donor site care due to its one-time use, since it does not require dressing changes, which, in turn, may also reduce subjective pain experienced by the patient.^34^

## Clinical Problem Addressed

Split-thickness skin grafting is a reconstructive procedure that is most commonly used for management of burn injuries. Skin harvesting creates a new partial thickness wound, a donor site that causes additional pain for the patient during the postoperative recovery. Therefore, and because wound healing complications, such as delayed healing and infections, are common on donor sites, the donor sites are problematic to treat. A dressing that would provide optimal healing, low costs, and minimal pain with few dressing changes would be a preferred choice for treatment of donor sites. This clinical study intends to present the performance of a new wood-derived NFC wound dressing as an effective dressing in treatment of skin graft donor sites.

## Materials and Methods

### Wound dressings

NFC wound dressing (FibDex^®^) was kindly provided by UPM-Kymmene Corporation, Finland. The dressing consists of non-woven fabric that is treated with NFC on both sides. The manufacturing process has been previously described.^30^ A commercially available polylactic acid-based copolymer dressing (Suprathel^®^; Polymedics Innovations GmbH, Germany) was used as a reference material.

### *In vitro* cytotoxicity of nanofibrillar cellulose dressing

The cytotoxic effect of NFC dressing was analyzed by using an XTT [(sodium-3′-(lphenylaminocarbonyl)-3,4-tetrazolium)-bis(4-methoxy-6-nitro) benzensulfonic acid hydrate] test based on the cleavage of the yellow tetrazolium salt XTT to form an orange water-soluble formazan dye by dehydrogenase activity in active mitochondria. First, NFC wound dressing was extracted under agitation for 24 ± 2 h in Dulbecco's modified Eagle's medium (DMEM; Gibco) supplemented with 10% fetal bovine serum (FBS) at 37 ± 1°C in compliance with the International Organization for Standardization (ISO) 10993-5 and 10993-12. The absorption capacity of NFC dressing was determined (25.6 mL extraction medium/g test item) and considered for the extraction. The final weigh/volume ratio in the assay was 0.2 g/mL above the absorption capacity, which corresponds to 100% extract concentration. As a negative control, polypropylene (Greiner; Art. No. 188.271, Lot-No. E16053QH) was extracted at a weigh/volume ratio of 1 g/5 mL medium. Latex Examination Gloves (VWR; Lot 2014-06 29980031) were used as a positive control and extracted at a surface/volume ratio of 6 cm^2^/mL of DMEM 10% FBS. A solvent control consisting of extraction vehicle (DMEM 10% FBS; Eurofins Munich, Lot No. 17011 3HIe) alone was treated in the same way as the treatment groups.

The cytotoxicity test was carried out with established L929 cells (ATCC No. CCL-1, NCTC clone 929 [connective tissue mouse], male, age 100 days, clone of strain L [DSMZ]) cultured in DMEM with 10% FBS-Gold (PAA Laboratories GmbH) at 37 ± 1°C and 5.0% CO_2_. The extract of the NFC dressing and the solvent control were diluted five times with DMEM 10% FBS at a ratio of 2:3, giving final concentrations of 13.2%, 19.8%, 29.6%, 44.4%, 66.7%, and 100%. One hundred microliters of the different dilutions or 100 μL of the controls were pipetted into three parallel cultures in an empty 96-well plate (Greiner). Subsequently, log-phase L929 cells were used for preparation of a single-cell suspension at a density of 8.0 × 10 cells/mL. Fifty microliters of this cell suspension were pipetted to a 96-well plate containing the extracts with the exception of the blanks. The cell culture plates were then incubated with the extracts for 68–72 h at 37 ± 1°C, 5.0% CO_2_. Then, 1–2 h before the end of the incubation period, 50 μL of the XTT labeling mixture (Roche Diagnostics; Cell Proliferation Kit II) was added to each well. The cells were incubated for further 1–2 h, and the plate was subsequently transferred to a microplate reader equipped with a 490-nm filter to read the absorbance (reference wavelength 630 nm).

### Patients

The clinical study was performed according to the Clinical Investigation of medical devices for human subjects, good clinical practice (ISO 14155:2011) at Helsinki Burn Centre, Helsinki University Hospital, Finland. The study was approved by the Research Ethics Committee at the Helsinki University Hospital (99/13/03/02/2014 and HUS/1166/2016), and it enrolled burn patients or patients requiring skin graft donor site treatment with exclusion criteria of pregnancy and age younger than 18 or older than 75 years. Subjects or their legal representatives were informed of procedures and provided written informed consent.

### Surgical procedure

A Zimmer^®^ air dermatome (Zimmer, Inc.) was used to harvest 6/1,000 inch (0.15 mm)–12/1,000 inch (0.30 mm) thick split-thickness skin grafts. The separate donor sites were covered with NFC dressing or with NFC and copolymer dressings as previously described,^30^ except in one patient whose single donor site was divided into two parts and treated with both dressings. Experimental dressings that were left in place for the entire treatment period were covered by Jelonet^®^ (Smith and Nephew, United Kingdom) and fixed with staples. When compared with the copolymer dressing, anatomically equivalent areas were chosen for donor sites. The dressings were randomly selected for the treatment of each donor site.

### Skin graft donor site treatment and observations

The healing time of the donor site was determined as the self-detachment day of the NFC dressing or the copolymer dressing. Both donor site materials behave similarly, detaching from the wound bed when new epithelium is regenerated.

Postoperatively, the experimental dressings on skin graft donor sites were checked by visual observation when changing the overlaying dressings at an interval of a few days on average on postoperative days (PODs) 4, 7, 10, 14, 20, and 28, or when clinically relevant (±1–3 days), until self-detachment. During observations, skin quality, the epithelialization percentage of the donor site skin, and the possible adverse effects were evaluated by a plastic surgeon. In addition, subjective pain experience was questioned from the patients using scale 0–10 (0 representing no pain and 10 the worst possible pain). Donor sites and wound dressings were photographed during the examinations throughout the clinical study period. Skin elasticity, viscoelasticity, and transepidermal water loss (TEWL) were measured after discharge, 1 and 6 months postoperatively. In addition, scar quality was evaluated by using the Patient and Observer Scar Assessment Scale (POSAS) that was translated to Finnish but not validated in Finnish language. The POSAS consists of two numerical scales: the patient and the observer scar assessment scale that scores six parameters on a 10-point rating scale, in which the highest score represents the worst imaginable scar.^35,36^

### Non-invasive measurements

Elasticity, viscoelasticity, and TEWL were measured from patients' epithelialized skin treated with NFC or copolymer dressings during follow-up examination at 1 and 6 months after commencement of the treatments using DermaLab^®^ Skinlab COMBO (Cortex Technology, Denmark), which is a reliable instrument for objective measurements of skin elasticity and TEWL.^37,38^ The elasticity of the skin was assessed in terms of elastic modulus and viscoelasticity. TEWL was expressed as g/m^2^/h to assess the epidermal barrier function. TEWL increases when the skin barrier is damaged and is, therefore, an important parameter to evaluate the efficiency of the human skin barrier. For measurements, a single (in case of TEWL measurement) or 4–5 (in case of elasticity measurements) successive readings were taken at the same site. Control measurements were taken on healthy, not-operated skin of the same subject at equal locations.

### Statistical analysis

Normal distribution of the data was tested for each parameter at each measurement point by the Shapiro-Wilk Test. Significant differences between independent data were analyzed by using Student's *t*-test (normal distribution) or Mann-Whitney U test (non-normal distribution) when comparing two groups or using one-way analysis of variance (normal distribution) or Kruskal-Wallis H test (non-normal distribution) when comparing more than two groups. Paired data were assessed by using paired *t*-test (normal distribution) or the Wilcoxon signed-ranks test (non-normal distribution). Values of *p* < 0.05 were considered statistically significant.

## Results

### Cytotoxicity of NFC wound dressing

The cytotoxicity of the NFC wound dressing was assessed by means of the XTT test using mouse cell line L929. NFC wound dressing was extracted under agitation, after which L929 cells were incubated with the following end concentrations of the extract: 13.2%, 19.8%, 29.6%, 44.4%, 66.7%, and 100%. The highest extract concentration corresponds to the ISO 10993-5 and 10993-12 described weight/volume ratio of 0.2 g/mL. The extraction procedure did not reveal any abnormalities in the extraction medium or the test item. No changes regarding clarity, color, and presence or absence of foreign material occurred in the extraction medium. The pH-value of the test extract was 7.5 (solvent control pH 7.5).

The results showed no relevant reduction of cell proliferation and/or cell viability. With the highest extract concentration (100%), the dehydrogenase activity was not reduced. Microscopically, no inhibition of cell growth and no cell lysis were observed at all extract concentrations used. The controls confirmed the validity of the study. Between the solvent control and the negative control, no relevant difference could be observed. The positive control showed a distinct reduction in cell viability and cell proliferation, as dehydrogenase activity was reduced to 1%.

### Patients' characteristics and the study course

**Figure 1.**
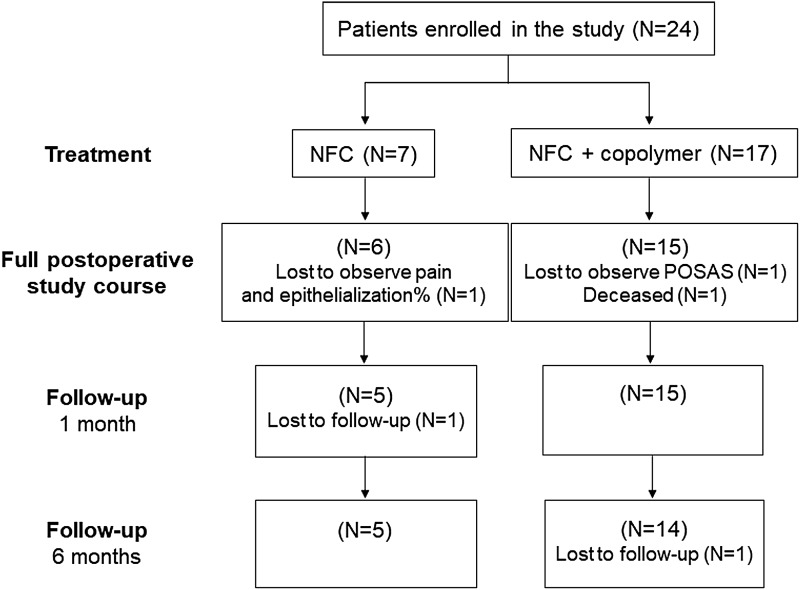
Flow chart of the clinical study. A total of 24 patients were enrolled in the study and were treated with NFC dressing, whereas 17 patients had an intra-individual comparison of NFC dressing and copolymer dressing. NFC, nanofibrillar cellulose; POSAS, Patient and Observer Scar Assessment Scale.

Progress through the study phases is presented in Fig. 1 as a flow diagram. We enrolled a total of 24 patients (patient no. 11–34), with 19 completing the full study course with NFC dressing treatment. An intra-individual comparison of NFC and copolymer dressings was performed in 17 patients. One patient was deceased on POD 22 and results of 4 other patients are lacking from the study due to failure to observe pain, POSAS, or the percentage of re-epithelialization or failure to attend follow-up appointments.

The baseline characteristics of all the enrolled patients are presented in Table 1. The average age of all the patients was 49 years ±18, range 21–74 years. Majority of the patients were male (67%). The total body surface area varied from 1% to 50%. Majority of the patients suffered from flame burns. All the patients were Caucasian. The total size of donor site(s) for a patient varied between 64 cm^2^ and 1,132 cm^2^ for the NFC dressing, and between 82.5 cm^2^ and 1,201 cm^2^ for the copolymer dressing.

**Table 1.** Patient demographics and clinical characteristics

Characteristics	NFC (*N* = 24)	Copolymer (*N* = 17)	*p*
Age, mean (SD)	49 (18)	49 (17)	1^*^
Gender F:M	8:16	4:13	—
TBSA (%), mean (SD)	16.5 (13.7)	20.0 (13.6)	0.42^*^
Etiology			
Flame burn	15 (62%)	15 (88%)	—
Scald burn	2 (8%)	0 (0%)	—
Electrical burn	1 (4%)	0 (0%)	—
Chemical injury	3 (13%)	0 (0%)	—
Contact with hot object	3 (13%)	2 (12%)	—
Comorbidities			
Abundant alcohol consumption	5 (21%)	3 (18%)	—
Asthma	3 (13%)	2 (12%)	—
Diabetes	3 (13%)	2 (12%)	—
Hypercholesterolemia	3 (13%)	1 (6%)	—
Hypertension	6 (25%)	3 (18%)	—
Obesity	4 (17%)	3 (18%)	—
Smoking	3 (13%)	2 (12%)	—
Location			
Back	3 (13%)	3 (18%)	—
Thigh	19 (79%)	13 (76%)	—
Scalp	1 (4%)	0 (0%)	—
Flank/stomach	1 (4%)	1 (6%)	—

^*^ Student's *t*-test.

NFC, nanofibrillar cellulose; SD, standard deviation; TBSA, total body surface area.

### Wound healing time and treatment

**Figure 2.**
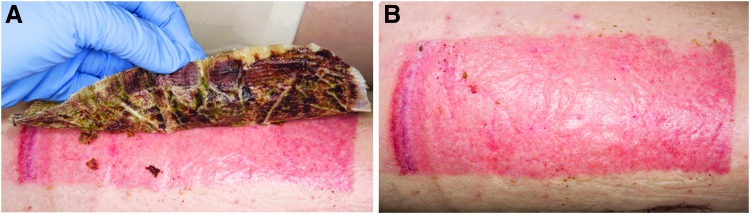
The detachment of NFC dressing from the donor site. **(A)** An experienced staff gently removes the dressing when the material is able to be detached without breaking the newly formed skin. **(B)** The epithelialized skin graft donor site after detachment of NFC dressing.

**Figure 3.**
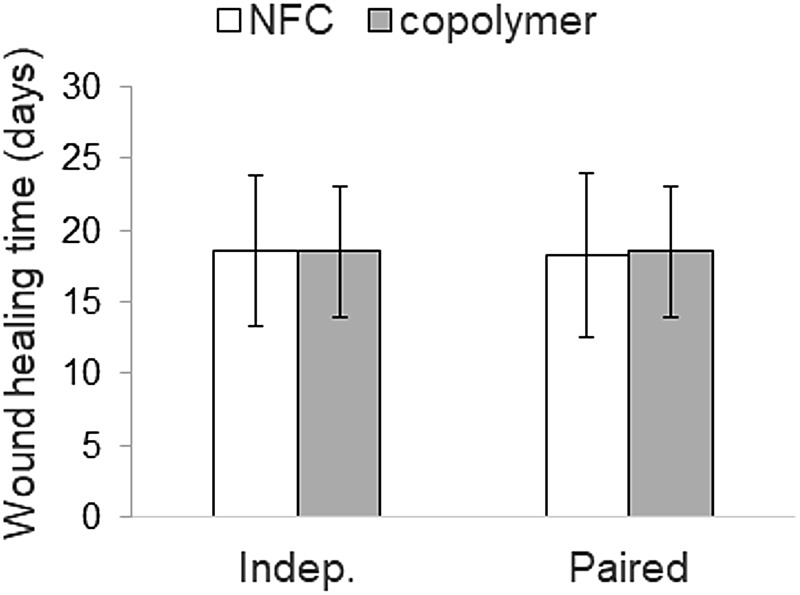
Wound healing time in days presented as mean (standard deviation). Indep., independent samples.

**Figure 4.**
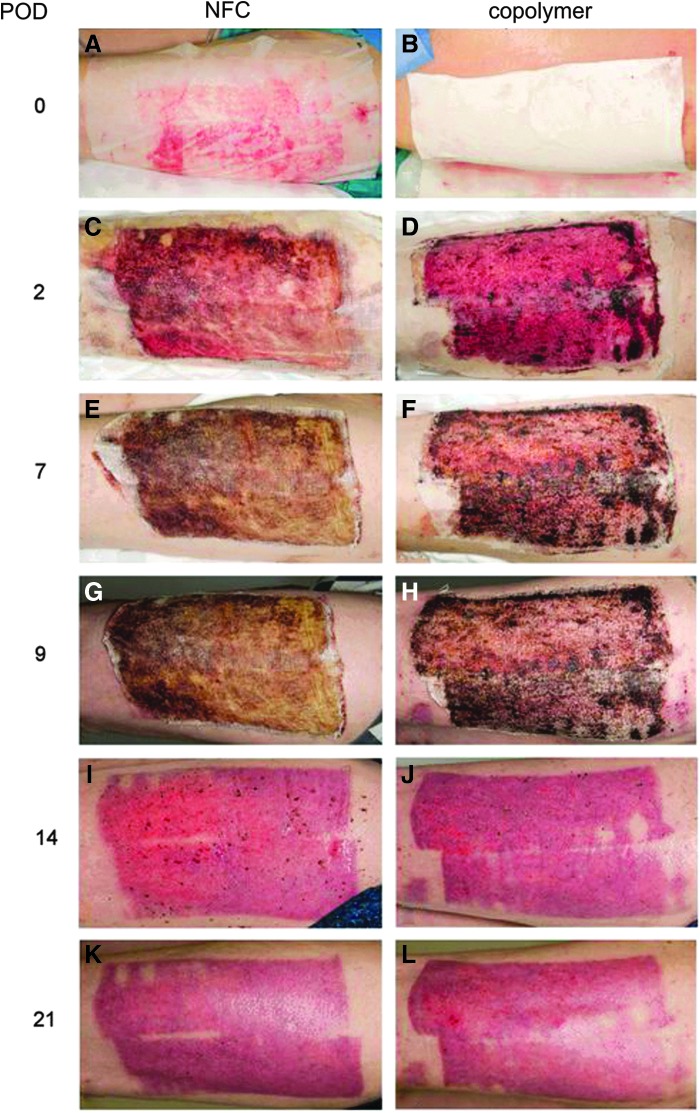
Skin graft donor site treatment with NFC and copolymer dressings of patient 28. **(A**, **B)** The skin graft donor site in operation. A transparent NFC dressing was placed on the left thigh **(A)**, and white copolymer dressing was placed on the right thigh **(B)**. **(C**, **D)** POD 2. Both dressings were dry and well attached to the donor site. **(E**, **F)** POD 7. Dressings were still dry and attached. **(G**, **H)** POD 9. Small pieces of both dressings were detached from the edges. **(I**, **J)** POD 14. Both dressings detached on the same day (POD 14), revealing an epithelialized donor site. **(K**, **L)** POD 21. One hundred percent of NFC dressing-treated donor site was epithelialized, whereas the epithelialization percentage for the copolymer-treated donor site was 97%. POD, postoperative day.

The healing time of the donor site was determined as the self-detachment day of the dressing. The detachment of NFC dressing is shown in Fig. 2. The mean healing time (Fig. 3) for NFC dressing (*N* = 24) with independent samples was 18.5 (±5.3) days and for the copolymer (*N* = 16) it was the same 18.5 (±4.6) days (*p* = 0.86, Mann-Whitney U test). In pairwise comparisons (*N* = 16), the mean healing time for NFC dressing was 18.3 (±5.7) days (*p* = 0.59 compared with copolymer, Wilcoxon signed-ranks test). Seven patients demonstrated the same healing time with both dressings, and six patients treated with NFC dressing showed 1–4 days shorter healing time compared with the copolymer. Figure 4 shows an example of the treatment of donor sites with NFC and copolymer dressings during the postoperative period.

### Adverse events observed during or after the treatment

Adverse events were observed in five patients treated with NFC dressing. Two of them were considered to be as a result of treatment with NFC dressing; in these cases, the dressing had partially slid off the donor site. The material was partly replaced with another dressing type. In comparison, the copolymer dressing had partly slid off the donor site in one patient. In two other patients, an infection was suspected on the donor site treated with NFC dressing. In these cases, the NFC dressing was partially replaced with other dressing. However, due to the condition of the patient, it is difficult to determine whether the infection occurred because of NFC dressing treatment or other clinical influencing factors. No infection occurred in the copolymer-treated donor site. In one patient, an infection was observed on both donor sites treated with NFC dressing and copolymer dressing.

Device deficiencies for NFC dressing were reported in two patients. In one, more hematoma formation was observed at the donor site treated with NFC dressing as compared with a similar donor site treated with the copolymer dressing for the same patient, but it did not cause any extra discomfort. For the same patient, small skin breaks were identified on the donor site treated with NFC dressing 1 month post-surgery, whereas the donor site treated with the copolymer dressing was intact. In one patient, the edges of adjacent NFC dressings moved slightly away from each other, thus revealing some wound surface, which was covered with other dressing material.

**Figure 5.**
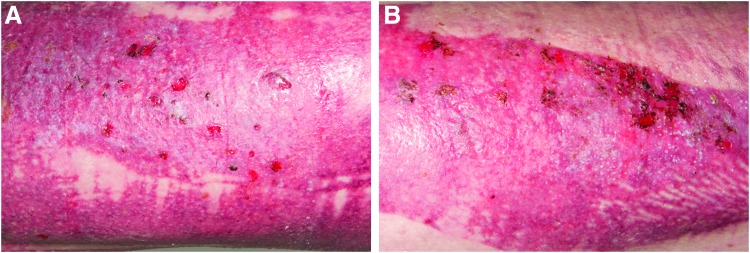
Skin graft donor sites after the treatment with NFC dressing on the left thigh **(A)** and the copolymer dressing on the right thigh **(B)** on the same patient on POD 28 after the detachment of the dressings (NFC on POD 15, copolymer on POD 17). Residual wounds were found on both donor sites.

After the treatment, that is, complete self-detachment of the dressing, residual wounds were observed in two patients treated with NFC dressing and in one patient treated with the copolymer dressing (Fig. 5).

### Assessments regarding skin epithelialization, pain, and scar characteristics

**Figure 6.**
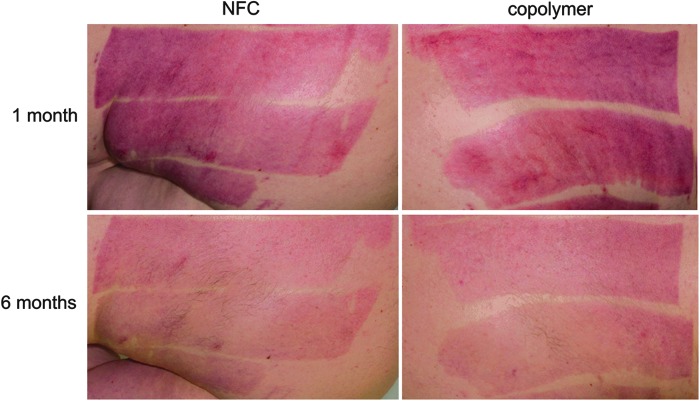
Epithelialization of the donor site skin at 1 and 6 months after treatment with NFC dressing and copolymer dressing.

We observed no statistically significant difference in the percentage of epithelialization between donor sites treated with NFC and copolymer dressings at POD 14 (46.4 ± 39.0% for NFC, 43.9 ± 40.0% for copolymer; *p* = 0.72, paired-samples *t*-test, *N* = 11) or at 1 or 6 months post-surgery (*N* = 13). At 1 month, donor site skin was not fully epithelialized in all patients (99.3 ± 1.2% for NFC, 99.5 ± 0.8% for copolymer; *p* = 0.34, paired-samples *t*-test), whereas at 6 months, 100% of each donor site was epithelialized (Fig. 6).

When evaluating the pain experience of the patients during the treatment and the follow-up, pain scores did not show any statistically significant difference between skin graft donor sites treated with the NFC dressing and those treated with the copolymer dressing at any of time points evaluated, even if there was a trend toward less pain in NFC dressing-treated donor sites (Table 2). The results of the translated but not validated POSAS revealed a significant difference between NFC and copolymer dressings for separate observations, including thickness in the Observer score at 1 month (*p* = 0.04) and vascularity in the Observer score at 6 months (*p* = 0.008), favoring NFC dressing (Supplementary Table S1). No significant differences were observed between NFC and copolymer dressings in the overall opinions by the Observer or Patient scale at 1 or 6 months (Table 2).

**Table 2.** Results of the pain score and the Patient and Observer Scar Assessment Scale

	POD 10–15	1 Month	6 Months
Pain			
NFC (*N* = 11)	0.55 (0.9)	0.82 (1.8)	0.27 (0.5)
copolymer (*N* = 11)	1.18 (2.2)	1.18 (1.8)	0.45 (0.9)
*p*	0.34^*^	0.17^*^	0.34^*^
POSAS observer score,^a^ overall opinion			
NFC (*N* = 13)	—	2.81 (0.8)	2.19 (0.7)
copolymer (*N* = 13)	—	2.85 (0.8)	2.31 (0.6)
*p*	—	0.66^**^	0.24^**^
POSAS patient score,^a^ overall opinion			
NFC (*N* = 13)	—	5.15 (2.9)	3.00 (1.6)^b^
copolymer (*N* = 13)	—	4.92 (3.0)	3.58 (2.2)^b^
*p*	—	0.47^**^	0.18^**^

Values are presented as mean (SD).

^a^ POSAS translated to Finnish, but not validated in Finnish language.

^b^ *N* = 12, scores of 1 patient are lacking.

^*^ Student's *t*-test.

^**^ Wilcoxon signed-ranks test.

*N*, number of patients; POD, postoperative day; POSAS, Patient and Observer Scar Assessment Scale.

### Scar quality measurements

Regarding the secondary outcomes measured using DermaLab during the patient follow-up, the paired data showed significantly smaller elasticity values in the NFC dressing-treated donor sites compared with the copolymer dressing-treated donor sites at 1 month (*N* = 14) with respect to both viscoelasticity (13.7 ± 3.4 MPa for NFC; 16.3 ± 5.3 MPa for copolymer; *p* = 0.02, Wilcoxon signed-ranks test) and elastic modulus (5.5 ± 1.0 MPa for NFC; 6.2 ± 1.6 MPa for copolymer; *p* = 0.01, paired-samples *t*-test) (Supplementary Fig. S1). However, no statistically significant differences were found between NFC and the copolymer dressing-treated donor sites at 1 month (*N* = 14) in terms of TEWL (29.8 ± 13.2 g/m^2^/h for NFC; 26.8 ± 9.7 g/m^2^/h for copolymer; *p* = 0.36, paired-samples *t*-test) or at 6 months (*N* = 12) in terms of viscoelasticity (12.4 ± 2.7 MPa for NFC; 12.9 ± 3.1 MPa for copolymer; *p* = 0.49, paired-samples *t*-test), elastic modulus (5.1 ± 0.8 MPa for NFC; 5.1 ± 0.9 MPa for copolymer; *p* = 0.83, paired samples *t*-test), or TEWL (9.0 ± 5.4 g/m^2^/h for NFC; 11.2 ± 7.4 g/m^2^/h for copolymer; *p* = 0.14, Wilcoxon signed-ranks test) in pairwise comparison (Supplementary Fig. S1).

The values of TEWL and elastic modulus from donor sites treated with NFC dressing and copolymer dressing differed significantly from the values of the healthy skin at 1 month, whereas viscoelasticity values at 1 month and all values at 6 months showed no differences between healthy skin and NFC dressing or the copolymer dressing-treated donor site skin (Supplementary Table S2). When comparing values recorded at 1 and 6 months, a significant improvement was observed with the NFC dressing regarding TEWL (*p* < 0.001) and with the copolymer dressing regarding TEWL (*p* = 0.01), viscoelasticity (*p* = 0.04), and elastic modulus (*p* = 0.047) between the time points (Supplementary Table S2).

## Discussion

This study was designed to compare the effects of NFC dressing on a synthetic polylactide-based copolymer dressing that is the most commonly used material to treat large skin graft donor sites at our burn center. In the treatment of skin graft donor sites, NFC dressing was found to provide equal wound healing time and epithelialization compared with the copolymer dressing. Importantly, however, the elasticity of epithelialized donor site skin was improved after treatment with NFC dressing, whereas no difference regarding TEWL was observed between NFC dressing and the copolymer dressing-treated donor sites. Further, scar quality assessed by using the translated but not validated POSAS suggested some advances in the use of NFC dressing over the copolymer dressing.

In light of the fact that split-thickness skin grafting can cause excess pain for the patient, several studies have shown the copolymer dressing to decrease pain scores in patients compared with other wound dressings.^31,33,39,40^ According to our results, pain scores reported for NFC dressing are similar to those reported for the copolymer dressing.

With regard to *in vitro* cytotoxicity testing, no particles in cytotoxic concentrations were released from NFC wound dressing under the given conditions used in this study, and as has been previously stated.^30^ During or after the treatment with NFC dressing, no major adverse events or allergic reactions were detected. In addition, no life-threatening complications were observed during the study. Wound healing is a complex process where the maturation of the skin after wound closure occurs slowly. The newly formed re-epithelialized skin is fragile and sensitive to mechanical interaction, and it may therefore easily break. In our study, superficial skin lesions, described as residual wounds, were observed in some patients after epithelialization of donor sites treated with both NFC dressing and copolymer dressing. In clinical use, NFC dressing is performed as a pliable wound dressing that adheres well to the wound bed.

The wound healing time was determined as the self-detachment day of the material, but the exact time for full epithelialization cannot be stated due to the nature of NFC dressing. During treatment, NFC dressing was covered by other dressings that kept it in place. NFC dressing was gently removed during the visual observation by experienced staff but only when the material was able to be detached without breaking the newly formed skin. In some patients, NFC dressing was removed piece by piece over several days, so that majority of the donor site had epithelialized whereas small pieces of the dressing were still attached. An equal procedure was used for the copolymer dressing. Therefore, epithelialization may have occurred before the final detachment of the material, which might explain the long healing times detected in this study compared with others.^41,42^ Further, wound healing time is affected by different comorbidities, such as abundant alcohol consumption, diabetes mellitus, obesity, or smoking, which impair the wound healing process.^43^ In this study, the majority of the enrolled patients suffered from one or more comorbidities.

The skin forms a protective barrier against pathogens, and an open wound always bears the risk of an infection. Wound infections observed during this study were not considered to be related to the use of NFC dressing. According to our results, use of NFC dressing does not appear to bear any more risk for infections than the copolymer dressing. Risk of infection may be enhanced in donor sites located in certain anatomical areas, or due to comorbidities of the patient.^43^ For the same reasons, and without a prominent infection, a wound may secrete an excessive amount of exudate that induces the detachment of the wound dressing. During the course of this study, this phenomenon was observed with both dressings.

One important aspect in wound treatment is the cost-effectiveness. The copolymer dressing has been rated as an expensive material compared with most of those used for treatment of skin graft donor sites^31,40^ and its acquisition costs are also quite high.^44^ NFC dressing is not yet commercially available, so the cost of a dressing has yet to be determined. However, consideration should be given to the fact that NFC dressing is produced from natural sustainable raw materials and it does not break down in the wound. In addition, it can be tuned to demand and manufactured in large sheets, a significant advantage over other dressings, which are often restricted to a maximum size due to their production process. Having bigger dressing sizes available makes treatment of extensive donor sites and wounds easier, as they may be covered by one dressing instead of several smaller dressings.

Regarding storage, NFC dressing is stored at room temperature (RT) and atmosphere, whereas the copolymer dressing requires storage in a refrigerator due to hydrolysis that may occur at RT. Other wound dressings may require even more specific storage conditions or additional materials for storage, such as storing in salt solution or in foil. Compared with these dressings, NFC dressing offered as a dry dressing is more ecological and does not require a cold chain for transportation.

The use of NFC in a wound dressing may allow modification and functionalization of the dressing, for example, by introducing drug molecules or proteins, thereby offering a wide variety of affordances for NFC dressing. Often, ionic silver is added in wound dressings, such as in BC- or CMC-based dressings, to induce antimicrobial properties.^16,27,45–47^ However, silver has been shown to have cytotoxic effects on cells.^48^ Recently, in contrast, Powell *et al.*^14^ showed that NFC originating from wood was able to inhibit bacterial growth, suggesting inherent anti-microbial properties of NFC. These properties would serve as an advanced feature for a wound dressing.

## Innovation

Skin graft donor site management requires special consideration, but no dressing type exists that is superior over other types of dressings regarding optimal healing. In this clinical study, the performance of NFC dressing was shown to be comparable to or even better than the reference copolymer dressing. NFC dressing is a totally new dressing created from wood-based material that is free from animal or human constituents, is safe to use, appears suitable for skin graft donor site treatment, and may result in better scar quality. Therefore, NFC dressing can be considered a promising material for future clinical applications.

**Key Findings**

- NFC dressing as treatment for skin graft donor sites performs comparable with the polylactide-based copolymer dressing
- NFC dressing requires no dressing changes, self-detaches as intact dressing after re-epithelialization, and does not degrade into tissue
- NFC dressing facilitates low pain experience
- NFC originates from nature, and it is a green sustainable product without animal or human-origin components

## Supplementary Material

Supplemental data

Supplemental data

## About the Authors

**Raili Koivuniemi, PhD** received her PhD in Life Sciences from the University of Helsinki (UH), Finland. She currently works as a postdoctoral researcher at the Division of Pharmaceutical Biosciences, UH with focus on stem cells and biomaterials for wound care applications. **Tiina Hakkarainen, MSc (Pharm.),** and **Jasmi Kiiskinen, MSc,** work as graduate students at the Division of Pharmaceutical Biosciences, UH. **Mika Kosonen, MSc (Tech.),** works as a production manager and **Kari Luukko, PhD (Tech.),** works as a quality and senior manager at UPM-Kymmene Corporation, Finland. **Jyrki Vuola, MD, PhD,** is a specialist plastic surgeon and a head of Helsinki Burn Centre, Finland. **Jussi Valtonen, MD** works as a specialist plastic surgeon at Helsinki Burn Centre. **Heli Kavola, MD, PhD** works as a specialist plastic surgeon at Helsinki University Central Hospital, Finland. **Marjo Yliperttula, PhD** is a principal investigator of Biopharmaceutics Research Group at the Division of Pharmaceutical Biosciences, UH.

## Abbreviations and Acronyms

BC: bacterial cellulose
CMC: carboxymethyl cellulose
DMEM: Dulbecco's modified Eagle's medium
FBS: fetal bovine serum
ISO: International Organization for Standardization
NFC: nanofibrillar cellulose
POD: postoperative day
POSAS: Patient and Observer Scar Assessment Scale
TEWL: transepidermal water loss
XTT: sodium-3′-(lphenylaminocarbonyl)-3,4-tetrazolium)-bis(4-methoxy-6-nitro) benzensulfonic acid hydrate

## References

[1] Uygur F, Evinc R, Ulkur E, Celikoz B Use of lyophilized bovine collagen for split-thickness skin graft donor site management. Burns 2008;34:1011–1014 18407418 10.1016/j.burns.2007.12.007

[2] Bradow BP, Hallock GG, Wilcock SP Immediate regrafting of the split thickness skin graft donor site assists healing. Plast Reconstr Surg Glob Open 2017;5:e1339 28607863 10.1097/GOX.0000000000001339 PMC5459646

[3] Murphy PS, Evans GR Advances in wound healing: a review of current wound healing products. Plast Surg Int 2012;2012:190436 22567251 10.1155/2012/190436 PMC3335515

[4] Kolakovic R, Peltonen L, Laukkanen A, Hirvonen J, Laaksonen T Nanofibrillar cellulose films for controlled drug delivery. Eur J Pharm Biopharm 2012;82:308–315 22750440 10.1016/j.ejpb.2012.06.011

[5] Valo H, Arola S, Laaksonen P, et al. Drug release from nanoparticles embedded in four different nanofibrillar cellulose aerogels. Eur J Pharm Sci 2013;50:69–77 23500041 10.1016/j.ejps.2013.02.023

[6] Paukkonen H, Kunnari M, Lauren P, et al. Nanofibrillar cellulose hydrogels and reconstructed hydrogels as matrices for controlled drug release. Int J Pharm 2017;532:269–280 28888974 10.1016/j.ijpharm.2017.09.002

[7] Bhattacharya M, Malinen MM, Lauren P, et al. Nanofibrillar cellulose hydrogel promotes three-dimensional liver cell culture. J Control Release 2012;164:291–298 22776290 10.1016/j.jconrel.2012.06.039

[8] Lauren P, Lou YR, Raki M, Urtti A, Bergstrom K, Yliperttula M Technetium-99m-labeled nanofibrillar cellulose hydrogel for in vivo drug release. Eur J Pharm Sci 2014;65:79–88 25245005 10.1016/j.ejps.2014.09.013

[9] Lou YR, Kanninen L, Kuisma T, et al. The use of nanofibrillar cellulose hydrogel as a flexible three-dimensional model to culture human pluripotent stem cells. Stem Cells Dev 2014;23:380–392 24188453 10.1089/scd.2013.0314 PMC3920753

[10] Therien-Aubin H, Wang Y, Nothdurft K, Prince E, Cho S, Kumacheva E Temperature-responsive nanofibrillar hydrogels for cell encapsulation. Biomacromolecules 2016;17:3244–3251 27615746 10.1021/acs.biomac.6b00979

[11] Zhang Y, Nypelö T, Salas C, Arboleda J, Hoeger IC, Rojas OJ Cellulose nanofibrils: from strong materials to bioactive surfaces. J Renew Mater 2013;1:195–211

[12] Chinga-Carrasco G, Syverud K Pretreatment-dependent surface chemistry of wood nanocellulose for pH-sensitive hydrogels. J Biomater Appl 2014;29:423–432 24713295 10.1177/0885328214531511 PMC4231171

[13] Lin N, Dufresne A Nanocellulose in biomedicine: current status and future prospect. Eur Polym J 2014;59:302–325

[14] Powell LC, Khan S, Chinga-Carrasco G, Wright CJ, Hill KE, Thomas DW An investigation of *Pseudomonas aeruginosa* biofilm growth on novel nanocellulose fibre dressings. Carbohydr Polym 2016;137:191–197 26686120 10.1016/j.carbpol.2015.10.024

[15] Sannino A, Demitri C, Madaghiele M Biodegradable cellulose-based hydrogels: design and applications. Materials 2009;2:353–373

[16] Fu L, Zhang J, Yang G Present status and applications of bacterial cellulose-based materials for skin tissue repair. Carbohydr Polym 2013;92:1432–1442 23399174 10.1016/j.carbpol.2012.10.071

[17] Alvarez OM, Patel M, Booker J, Markowitz L Effectiveness of a biocellulose wound dressing for the treatment of chronic venous leg ulcers: results of a single center randomized study involving 24 patients. Wounds 2004;16:224–233

[18] Portal O, Clark WA, Levinson DJ Microbial cellulose wound dressing in the treatment of nonhealing lower extremity ulcers. Wounds 2009;21:1–3 25904579

[19] Solway DR, Consalter M, Levinson DJ Microbial cellulose wound dressing in the treatment of skin tears in the frail elderly. Wounds 2010;22:17–19 25901459

[20] Bodin A, Ahrenstedt L, Fink H, Brumer H, Risberg B, Gatenholm P Modification of nanocellulose with a xyloglucan-RGD conjugate enhances adhesion and proliferation of endothelial cells: implications for tissue engineering. Biomacromolecules 2007;8:3697–3704 18031014 10.1021/bm070343q

[21] Czaja W, Krystynowicz A, Bielecki S, Brown RM Jr Microbial cellulose—the natural power to heal wounds. Biomaterials 2006;27:145–151 16099034 10.1016/j.biomaterials.2005.07.035

[22] Vandamme EJ, De Baets S, Vanbaelen A, Joris K, De Wulf P Improved production of bacterial cellulose and its application potential. Polym Degrad Stab 1998;59:93–99

[23] Bäckdahl H, Helenius G, Bodin A, et al. Mechanical properties of bacterial cellulose and interactions with smooth muscle cells. Biomaterials 2006;27:2141–2149 16310848 10.1016/j.biomaterials.2005.10.026

[24] Stumpf TR, Yang X, Zhang J, Cao X In situ and ex situ modifications of bacterial cellulose for applications in tissue engineering. Mater Sci Eng C Mater Biol Appl 2018;82:372–383 29025671 10.1016/j.msec.2016.11.121

[25] Capanema NSV, Mansur AAP, de Jesus AC, Carvalho SM, de Oliveira LC, Mansur HS Superabsorbent crosslinked carboxymethyl cellulose-PEG hydrogels for potential wound dressing applications. Int J Biol Macromol 2017;106:1218–1234 28851645 10.1016/j.ijbiomac.2017.08.124

[26] Kono H Characterization and properties of carboxymethyl cellulose hydrogels crosslinked by polyethylene glycol. Carbohydr Polym 2014;106:84–93 24721054 10.1016/j.carbpol.2014.02.020

[27] Barnea Y, Weiss J, Gur E A review of the applications of the hydrofiber dressing with silver (Aquacel Ag) in wound care. Ther Clin Risk Manag 2010;6:21–27 20169033 PMC2817785

[28] Masella PC, Balent EM, Carlson TL, Lee KW, Pierce LM Evaluation of six split-thickness skin graft donor-site dressing materials in a swine model. Plast Reconstr Surg Glob Open 2014;1:e84 25289278 10.1097/GOX.0000000000000031 PMC4174104

[29] Dornseifer U, Lonic D, Gerstung TI, et al. The ideal split-thickness skin graft donor-site dressing: a clinical comparative trial of a modified polyurethane dressing and aquacel. Plast Reconstr Surg 2011;128:918–924 21681125 10.1097/PRS.0b013e3182268c02

[30] Hakkarainen T, Koivuniemi R, Kosonen M, et al. Nanofibrillar cellulose wound dressing in skin graft donor site treatment. J Control Release 2016;244:292–301 27491880 10.1016/j.jconrel.2016.07.053

[31] Schwarze H, Kuntscher M, Uhlig C, et al. Suprathel, a new skin substitute, in the management of donor sites of split-thickness skin grafts: results of a clinical study. Burns 2007;33:850–854 17493762 10.1016/j.burns.2006.10.393

[32] Lasprilla AJ, Martinez GA, Lunelli BH, Jardini AL, Filho RM Poly-lactic acid synthesis for application in biomedical devices—a review. Biotechnol Adv 2012;30:321–328 21756992 10.1016/j.biotechadv.2011.06.019

[33] Schwarze H, Kuntscher M, Uhlig C, et al. Suprathel, a new skin substitute, in the management of partial-thickness burn wounds: results of a clinical study. Ann Plast Surg 2008;60:181–185 18216512 10.1097/SAP.0b013e318056bbf6

[34] Upton D and Andrews A The impact of stress at dressing change in patients with burns: a review of the literature on pain and itching. Wounds 2014;26:77–82 25860332

[35] Van de Kar AL, Corion LU, Smeulders MJ, Draaijers LJ, van der Horst CM, van Zuijlen PP Reliable and feasible evaluation of linear scars by the Patient and Observer Scar Assessment Scale. Plast Reconstr Surg 2005;116:514–522 16079683 10.1097/01.prs.0000172982.43599.d6

[36] Draaijers LJ, Tempelman FR, Botman YA, et al. The patient and observer scar assessment scale: a reliable and feasible tool for scar evaluation. Plast Reconstr Surg 2004;113:1960–1965; discussion 1966–1967. 15253184 10.1097/01.prs.0000122207.28773.56

[37] Gankande TU, Duke JM, Danielsen PL, DeJong HM, Wood FM, Wallace HJ Reliability of scar assessments performed with an integrated skin testing device-the DermaLab combo(®). Burns 2014;40:1521–1529 24630817 10.1016/j.burns.2014.01.025

[38] Fluhr JW, Feingold KR, Elias PM Transepidermal water loss reflects permeability barrier status: validation in human and rodent in vivo and ex vivo models. Exp Dermatol 2006;15:483–492 16761956 10.1111/j.1600-0625.2006.00437.x

[39] Hundeshagen G, Collins VN, Wurzer P, et al. A prospective, randomized, controlled trial comparing the outpatient treatment of pediatric and adult partial-thickness burns with Suprathel or Mepilex Ag. J Burn Care Res 2017;39:261–267 10.1097/BCR.0000000000000584 PMC5700875 28557869

[40] Markl P, Prantl L, Schreml S, Babilas P, Landthaler M, Schwarze H Management of split-thickness donor sites with synthetic wound dressings: results of a comparative clinical study. Ann Plast Surg 2010;65:490–496 20841998 10.1097/SAP.0b013e3181d37624

[41] Kazanavičius M, Cepas A, Kolaityte V, Simoliuniene R, Rimdeika R The use of modern dressings in managing split-thickness skin graft donor sites: a single-centre randomized controlled trial. J Wound Care 2017;26:281–291 28598760 10.12968/jowc.2017.26.6.281

[42] Haith LR, Stair-Buchmann ME, Ackerman BH, et al. Evaluation of Aquacel Ag for autogenous skin donor sites. J Burn Care Res 2015;36:602–606 25501785 10.1097/BCR.0000000000000212

[43] Guo S, DiPietro LA Factors affecting wound healing. J Dent Res 2010;89:219–229 20139336 10.1177/0022034509359125 PMC2903966

[44] Fischer S, Kremer T, Horter J, et al. Suprathel^®^ for severe burns in the elderly: case report and review of the literature. Burns 2016;42:e86–e92 27233678 10.1016/j.burns.2016.05.002

[45] Rajwade JM, Paknikar KM, Kumbhar JV Applications of bacterial cellulose and its composites in biomedicine. Appl Microbiol Biotechnol 2015;99:2491–2511 25666681 10.1007/s00253-015-6426-3

[46] Hebeish A, Hashem M, El-Hady MM, Sharaf S Development of CMC hydrogels loaded with silver nano-particles for medical applications. Carbohydr Polym 2013;92:407–413 23218313 10.1016/j.carbpol.2012.08.094

[47] Jung R, Kim Y, Kim HS, Jin HJ Antimicrobial properties of hydrated cellulose membranes with silver nanoparticles. J Biomater Sci Polym Ed 2009;20:311–324 19192358 10.1163/156856209X412182

[48] Burd A, Kwok CH, Hung SC, et al. A comparative study of the cytotoxicity of silver-based dressings in monolayer cell, tissue explant, and animal models. Wound Repair Regen 2007;15:94–104 17244325 10.1111/j.1524-475X.2006.00190.x

