# Supplementary material for: Clinical Study of Nanofibrillar Cellulose Hydrogel Dressing for Skin Graft Donor Site Treatment
Source: Adv Wound Care (New Rochelle). 2020 Feb 7;9(4):199–210. doi: 10.1089/wound.2019.0982 (PMC7047117; doi:10.1089/wound.2019.0982)
Supplement: Supplemental data [file Supp_FigS1-TableS1.pdf]

Supplementary Data

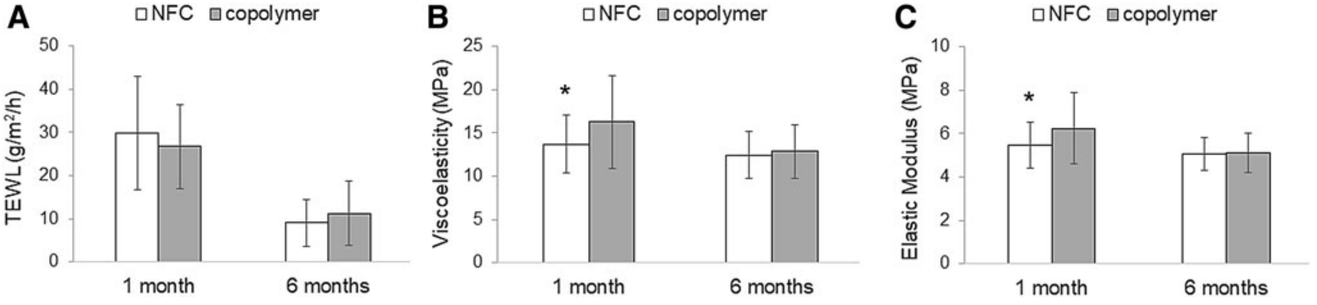

Supplementary Figure S1. Pairwise comparison of skin characteristics after treatment with NFC or copolymer dressings during follow-up at 1 and 6 months. Values are presented as mean (standard deviation). **(A)** TEWL. **(B)** Viscoelasticity. **(C)** Elastic modulus. *$p < 0.05$. NFC, nanofibrillar cellulose; TEWL, transepidermal water loss.

Supplementary Table S1. *Scores of the Patient and Observer Scar Assessment Scale*

	NFC	*Copolymer*	*Improvement (%)*	p
Observer scale: 1 month (*N*=13)				
Vascularity	4.23 (1.3)	4.31 (1.11)	1.8	0.14
Pigmentation	1.54 (1.13)	1.46 (0.97)	–5.3	0.47
Thickness	2.08 (0.86)	2.38 (0.87)	12.9	0.04*
Relief	2.62 (0.96)	2.77 (0.93)	5.6	0.11
Pliability	2.69 (0.85)	2.69 (1.32)	0	1
Surface area	1.00 (0)	1.00 (0)	0	1
Overall opinion	2.81 (0.75)	2.85 (0.83)	1.4	0.66
Observer scale: 6 months (*N*=13)				
Vascularity	2.46 (0.52)	2.85 (0.69)	13.5	0.008*
Pigmentation	1.92 (1.12)	2.23 (0.83)	13.8	0.11
Thickness	1.62 (0.87)	1.92 (1.04)	16.0	0.18
Relief	1.77 (1.01)	1.81 (1.15)	2.1	0.48
Pliability	2.00 (1.00)	1.92 (0.76)	–4.0	0.35
Surface area	1.23 (0.44)	1.23 (0.60)	0	1
Overall opinion	2.19 (0.66)	2.31 (0.60)	5.0	0.24
Patient scale: 1 month (*N*=13)				
Pain	1.81 (1.55)	2.35 (2.01)	23.0	0.23
Itching	2.23 (1.74)	2.50 (2.10)	10.8	0.35
Color[a]	8.30 (2.16)	8.40 (2.01)	1.2	0.66
Pliability[b]	3.09 (2.55)	3.27 (2.28)	5.6	0.29
Thickness[a]	3.05 (1.54)	3.70 (1.83)	17.6	0.14
Relief[a]	4.60 (2.84)	4.55 (2.77)	–1.1	0.32
Overall opinion	5.15 (2.94)	4,92 (2.96)	–4.7	0.47
Patient scale: 6 months (*N*=12)				
Pain	1.00 (0)	1.17 (0.58)	14.3	0.32
Itching	1.67 (0.89)	2.00 (1.54)	16.7	0.32
Color[c]	5.27 (2.57)	5.45 (2.16)	3.3	0.29
Pliability	1.83 (1.11)	2.33 (2.10)	21.4	0.32
Thickness[c]	1.64 (0.81)	2.36 (2.01)	30.8	0.11
Relief[c]	2.00 (1.18)	2.54 (2.16)	21.4	0.18
Overall opinion	3.00 (1.60)	3.58 (2.19)	16.3	0.18

Values for NFC dressing and the copolymer dressing are presented as mean (SD).

[a]Values of three patients lacking.

[b]Values of two patients lacking.

[c]Values of one patient lacking.

*Significant difference (Wilcoxon signed-ranks test)

N, number of patients; NFC, nanofibrillar cellulose; SD, standard deviation.
