# Supplementary material for: Clinical Study of Nanofibrillar Cellulose Hydrogel Dressing for Skin Graft Donor Site Treatment
Source: Adv Wound Care (New Rochelle). 2020 Feb 7;9(4):199–210. doi: 10.1089/wound.2019.0982 (PMC7047117; doi:10.1089/wound.2019.0982)
Supplement: Supplemental data [file Supp_TableS2.pdf]

Supplementary Table S2. *Characteristics of the epithelialized skin during follow-up*

	1 Month	*6 Months*[a]	p
TEWL ($g/m^2/h$)			
NFC ($N=19$)	28.0 (11.8)	8.6 (4.7)	<0.001*
Copolymer ($N=14$)	26.8 (9.7)	11.2 (7.4)	0.01*
Healthy skin ($N=19$)	6.2 (3.7)	8.1 (4.5)	—
p	<0.001**	0.81***	
Viscoelasticity (MPa)			
NFC ($N=19$)	14.6 (4.2)	12.2 (2.3)	0.12*
Copolymer ($N=14$)	16.3 (5.3)	12.9 (3.1)	0.04*
Healthy skin ($N=19$)	10.4 (3.2)	11.4 (3.3)	—
p	0.50***	0.42**	
Elastic modulus (MPa)			
NFC ($N=19$)	5.7 (1.2)	5.1 (0.7)	0.12†
Copolymer ($N=14$)	6.2 (1.6)	5.1 (0.9)	0.047†
Healthy skin ($N=19$)	4.6 (0.9)	4.8 (1.0)	—
p	0.002**	0.47**	

Values are presented as mean (SD).
[a]$N=17$ for NFC, $N=12$ for copolymer.
*Wilcoxon signed-ranks test; 1 month vs. 6 months
**One-way analysis of variance; comparison of the three groups.
***Kruskal-Wallis H test; comparison of the three groups.
†Paired-samples *t*-test; 1 month vs. 6 months.
TEWL, transepidermal water loss.
